# What can we learn from adherence data in adolescents participating in a clinical trial?

**DOI:** 10.1186/1687-9856-2015-S1-P7

**Published:** 2015-04-28

**Authors:** Jemma Jay Angela Anderson, Catherine Leggett, Jenny Couper, Alexia Peña

**Affiliations:** 1 Women's and Children's Hospital, Endocrine and Diabetes Centre, North Adelaide, SA, Australia; 2 The University of Adelaide, Discipline of Paediatrics, North Adelaide, SA, Australia; 3 SA Pharmacy Women's and Children's Hospital, North Adelaide, SA, Australia

## Introduction

Treatment adherence during adolescence is challenging. Limited data exist for the rate of medication adherence in adolescents.

## Aim

To evaluate medication adherence using two different methods (electronic monitoring system and tablet count) in adolescents with Type 1 Diabetes (T1D) participating in an RCT.

## Method

Medication adherence was assessed in 54 T1D adolescents (age 14±2.3y, 26 males) enrolled in a 1 year RCT to receive metformin or placebo [1].

Adherence was assessed using tablet count and prospective electronic monitoring using Medication Event Monitoring System caps (MEMS, AARDEX group, LTD Sion, Switzerland), which recorded episodes of bottle opening for each participant. These data were then compared to prescribed doses.

Adequate adherence was defined as ≥ 80% of prescribed doses taken over a defined period. Adherence was assessed after allowing a 3-month run-in period to account for dose titration and minimise potential for reactivity associated with initial adherence monitoring. Data were reported from 3-6 months (short-term) and from 6-12 months (long-term).

## Results

Adolescents had mean T1D duration 5.9±4.2y, median HbA1c 8.5 (6.3-11.6)%. 26 used CSII.

MEMS adherence data for short and long-term use was available for 53 and 48 participants respectively. Adequate adherence using MEMS was 47% (25/53) short-term and 35 (17/48) long-term. Median (IQR 25-75%) adherence was 79 (46.6-88.7%) short-term and 67 (43.4-86.8%) long-term.

Tablet count adherence data for short and long-term use was available for 35 and 42 participants respectively. Adequate adherence using tablet count was 63% (22/35) short-term and 45% (19/42) long-term. Median (IQR 25-75%) was 84 (70.8-90.1%) short-term and 77 (61.7-86.8%) long-term.

There was no statistically significant difference in adherence between the two methods used p=0.22 (short-term) and p=0.07 (long-term). Adolescents who were adherent in the short-term by MEMS were more likely to have a longer diabetes duration (7.2 vs 4.8 years, p=0.03).

## Conclusion

Adolescent adherence to intervention in this clinical trial was suboptimal as shown by both electronic monitoring and tablet count. This finding reinforces the importance of parental involvement in treatment regimens in this population.
